# Miscellaneous

**Published:** 1857-06

**Authors:** 


### Miscellaneous.

### GOLD SOLDER—18 CARAT

To each dwt. gold coin, add $5\frac{1}{2}$ grs. copper and silver, in equal parts, and $1\frac{1}{2}$ grs. pure zinc, (not plate) to be put in after the other metals are well mixed, and then turn off immediately and remelt.

This solder will flow on good silver plate quite freely.

L. P. Haskell.

The above solder is, with some, quite a favorite. Its true carat, however, is 17, not 18. But if the zinc all burns out, as is maintained by some, its standard will be nearly 18. We don't like brittle alloys for soldering gold, and, hence, we never add zinc, except for experiment. We hope all will try for themselves and not condemn it because we do.—Ed.

### AMERICAN DENTAL CONVENTION.

The third annual meeting of the "American Dental Convention" will be held in the City of Boston, on Tuesday, the 1st day of August, next, at 12 o'clock, M.

This Convention is open to "*all practicing Dentists*," and it is hoped that the profession will be largely represented.

State and local societies are expected to send one or more delegates.

At the last annual meeting, the following resolution was adopted:

*Resolved*, That the corresponding Secretary request all persons having any thing new and useful to the Dental Profession to present it at the next meeting.

W. W. ALLPORT,
Chicago, March 16, 1857. Corresponding Secretary.

---

### CORRECTION.

The following we clip from the "Dental Reporter," published by Jno. T. Toland. We are sorry that a correction was needed, and would be glad if we could get a condensed report that would misrepresent but one member, and would willingly be that member ourself. The last sentence may strike some as not *very* modest for a *young* man, but, with those who know that Dr. O. '*ails* from *Hengland*, it will all be right.

### ERROR IN THE DENTAL REGISTER OF THE WEST.

"Dr. Osmond requests us to state that, owing to the blunder of the individual who reported the proceedings of the Mississippi Valley Association, he was made to say, in the course of a debate on the causes of decay of the dental organs, that "In most American constitutions, *muriatic acid* is produced in too great a quantity to be consumed by the lungs." He trusts that those who are acquainted with him consider that he is sufficiently "*posted*" in Physiology, to be incapable of stating such a gross error. The report should read, "In most American constitutions, *carbon is taken* in too great a quantity to be consumed by the lungs." As to the *muriatic acid*, it is the acid of the gastric juice, according to our best Physiological authorities; combined with pepsin, it acts upon the nitrogenized substances, and also acts as a solvent of the phosphate of lime, consequently, when it is neutralized by alkalies, it loses this latter power; hence a deficiency of that salt, and a tendency to caries. This was discussed at length, but not reported.

Taken altogether, the report was not only meagre, but partial. Ideas advanced by younger members of the profession, in accordance with the recent discoveries in physiology, were either meagerly reported or omitted, to make room for the *lead* of one of the "great guns," which has been so often re-cast and shot over again, that it now contains considerable *dross*."

---

**MODEL ADVERTISEMENT.**

The following, which we clip from the "Indianapolis Daily Journal," may serve as a guide to the uninitiated. Many are able to advertise after this manner, without a "model;" but, to place all on equal footing, we insert it for the benefit of "whom it may concern:"

ANOTHER DENTIST, AS IS A DENTIST.

S. C. BLARNEY, M. D., D. D. S., F. R. S., 1001—Avails himself of the privileges offered by the free and unrestrained press, of informing the citizens of Indianapolis and Stringtown that he has *actually arrived*, and taken rooms for the practice of his profession. Having had the experience of a large and *unsuccessful* practice among the Hottentots, for the last seven years, (more or less) combined with the determination to excel, does not hesitate, (nor never did,) to say that all persons who need Dental operations, and wish them executed in the most *unprecedented manner*, *without the inconvenience of trying at all*, may feel the fullest and most entire *confidence* in him as a Dentist of established reputation, "hard to beat." Incorruptible porcelain, Hippopotamus, Calves, Donkeys, Human, and *all* other teeth inserted on Gold, Silver, Platina, Copper and Brass, *on suction at that*, with his (mine of course,) improved continuous gum, *colored to death*, from one to a thousand. The beauty of the production can be imagined. Every operation for the preservation and beauty of the Human and inhuman teeth, will be performed in the latest improved Hottentot style, and at prices which cannot fail to ruin.

All work *warranted* to perform without the aid of steam, wind or gas. The best of references given and required.

Office hours from 7 A. M. to 6 P. M., Sundays not excepted.

P. S. Dr. B. would hardly have given Indianapolis the benefit of his talents, had it not been for the high price of board among the Hottentots, and *too much business*, which was injuring his health."

